# Integrity of cortical perineuronal nets influences corticospinal tract plasticity after spinal cord injury

**DOI:** 10.1007/s00429-013-0701-9

**Published:** 2014-01-31

**Authors:** C. Orlando, O. Raineteau

**Affiliations:** Brain Research Institute, University of Zurich/ETH, Winterthurerstrasse 190, 8057 Zurich, Switzerland

**Keywords:** Extracellular matrix, Chondroitin sulphate proteoglycans, Chondroitinase, Parvalbumin, Plasticity

## Abstract

The rapid decline of injury-induced neuronal circuit remodelling after birth is paralleled by the accumulation of chondroitin sulphate proteoglycans (CSPGs) in the extracellular matrix, culminating with the appearance of perineuronal nets (PNNs) around parvalbumin-expressing GABAergic interneurons. We used a spinal cord injury (SCI) model to study the interplay between integrity of PNN CSPGs in the sensorimotor cortex, anatomical remodelling of the corticospinal tract (CST) and motor recovery in adult mice. We showed that thoracic SCI resulted in an atrophy of GABAergic interneurons in the axotomized hindlimb cortex, as well as in a more widespread downregulation of parvalbumin expression. In parallel, spontaneous changes in the integrity of CSPG glycosaminoglycan (GAG) chains associated with PNNs occurred at the boundary between motor forelimb and sensorimotor hindlimb cortex, a region previously showed to undergo reorganization after thoracic SCI. Surprisingly, full digestion of CSPG GAG chains by intracortical chondroitinase ABC injection resulted in an aggravation of motor deficits and reduced sprouting of the axotomized CST above the lesion. Altogether, our data show that changes in the expression pattern of GABAergic markers and PNNs occur in regions of the sensorimotor cortex undergoing spontaneous reorganization after SCI, but suggest that these changes have to be tightly controlled to be of functional benefit.

**Electronic supplementary material:**

The online version of this article (doi:10.1007/s00429-013-0701-9) contains supplementary material, which is available to authorized users.

## Introduction

An incomplete spinal cord injury (SCI) is followed by spontaneous anatomical and functional reorganization of motor circuits at multiple levels of the neuraxis (Oudega and Perez 2012; Raineteau and Schwab 2001). After a thoracic lesion, axotomized hindlimb corticospinal neurons located at the boundary between motor forelimb (mFL) and sensorimotor hindlimb (smHL) cortical areas are functionally incorporated into the sensorimotor circuits of the unaffected forelimb (Fouad et al. 2001; Ghosh et al. 2009, 2010). In parallel, sprouting of axotomized corticospinal fibres occurs in the spinal cord, both rostrally and caudally to the site of injury (Fouad et al. 2001; Weidner et al. 2001; Ghosh et al. 2009; Courtine et al. 2008), leading to the formation of new functional circuits and the partial re-establishment of supraspinal motor control (Bareyre et al. 2004; Fouad et al. 2001; Ghosh et al. 2010). These compensatory responses are however limited and not sufficient to support complete functional recovery. Chondroitin sulphate proteoglycans (CSPGs), perineuronal nets (PNNs) and parvalbumin-expressing (PV+) interneurons are some of the main factors which limit experience-dependent remodelling of adult neuronal circuits. CSPGs are the main components of the mature extracellular matrix (ECM) where they are diffusely expressed or aggregated as PNNs mainly around PV+ interneurons. In the cortex, PNN appearance regulates the maturation of PV inhibitory circuits and correlates with the gradual closure of the critical period for plasticity (Pizzorusso et al. 2002; Sale et al. 2007; Makara et al. 2007; Beurdeley et al. 2012; Dityatev et al. 2007). Chondroitinase ABC (ChABC)-mediated digestion of CSPGs and PNNs in animal model of SCI promotes axonal regeneration at the site of injury (Bradbury et al. 2002; Carter et al. 2008; Chau et al. 2004; Moon et al. 2001; Cafferty et al. 2007) and enhances functional sprouting of both intact and injured pathways rostral and caudal to the lesion site (Barritt et al. 2006; Cafferty et al. 2008; Massey et al. 2006; Starkey et al. 2012; Tom et al. 2009; Bradbury et al. 2002; Caggiano et al. 2005; Fouad et al. 2005; Tester and Howland 2008). In the adult visual cortex, ChABC-mediated PNN digestion restores ocular dominance plasticity (Pizzorusso et al. 2002). In the same system, pharmacological reduction of GABAergic transmission leads to a decrease in the expression of cortical PNNs and reactivation of ocular dominance plasticity (Harauzov et al. 2010). In animal models of stroke, spontaneous downregulation of PNN expression occurs in cortical area (i.e. peri-infarct and remote areas) known to undergo compensatory remodelling of neuronal networks (Bidmon et al. 1998; Hobohm et al. 2005). Thus, CSPGs, PNNs and PV+ cells act in concert to restrict anatomical and functional plasticity of the injured adult CNS.

In this study, we investigated the consequences of a thoracic SCI on the expression of GABAergic markers and PNN CSPGs across the mouse sensory and motor cortical areas which are known to undergo sustained reorganization after SCI (Ghosh et al. 2009, 2010). We showed that SCI induced a localized and layer-specific modulation of the cortical inhibitory system and led to changes in the integrity of PNN CSPGs at the boundary between the motor forelimb (mFL) and the sensorimotor hindlimb (smHL) cortical areas. Intracortical injection of ChABC to further digest CSPG GAG chains did however not promote functional recovery. Contrariwise, it prevented spontaneous sprouting of the axotomized CST neurons in the cervical spinal cord and resulted in a transient, limb-specific aggravation of motor deficits, suggesting that these cortical changes must be tightly controlled to be of functional benefit.

## Methods

C57BL/6 and GAD67-GFP female mice (mean age 2.5 months, *n* = 54) were used in this study. All surgical procedures and experimental manipulations were performed in agreement with Canton of Zurich Veterinary Office guidelines.

### Experimental design

Mice were divided into seven experimental groups as indicated in Table 1. Heterozygous knock-in mice expressing an enhanced green fluorescent protein (EGFP) under the glutamic decarboxylase GAD67 genomic regulatory sequences (i.e. GAD67^+/EGFP^ mice) were used in groups 1 and 2 to study the effects of thoracic SCI on the expression of cortical GABAergic markers and PNNs. In this knock-in mouse line, 95 % of GABAergic cells express GFP in a spatially and temporally regulated manner (Tamamaki et al. 2003). These animals were euthanized 2 weeks after SCI. Wild-type C57BL/6 mice were used in groups 3–7. Mice of group 3 were euthanized at 2, 7 and 14 days after cortical injection of ChABC to estimate the diffusion of the enzyme. Mice of groups 4–7 were used to address the extent of locomotor recovery after SCI and the role of cortical CSPG GAG chains digestion on the spontaneous cervical sprouting of axotomized hindlimb corticospinal neurons. Mice received a cortical injection of ChABC (groups 5 and 7) or of the control enzyme penicillinase (Pase; groups 4 and 6). Groups 6 and 7 underwent in addition a dorsal thoracic hemisection. All mice were tested for locomotor recovery ability and received an anterograde tracer injection at 28 days post-injury (dpi) to trace their CST. All mice from groups 4–7 were euthanized 6 weeks after injury.

**Table 1:** Experimental groups

	Strain	Enzymatic treatment	Injury	Motor test	Tracing
Group 1 (*n* = 4)	GAD67^+/EGFP^	n.p.	n.p.	n.p.	n.p.
Group 2 (*n* = 7)	GAD67^+/EGFP^	n.p.	T8	n.p.	Retrograde DY
Group 3 (*n* = 12)	C57BL/6	ChABC/vehicle	n.p.	n.p.	n.p.
Group 4 (*n* = 6)	C57BL/6	Pase	n.p.	BMS/grid	Anterograde BDA
Group 5 (*n* = 7)	C57BL/6	ChABC	n.p.	BMS/grid	Anterograde BDA
Group 6 (*n* = 9)	C57BL/6	Pase	T8	BMS/grid	Anterograde BDA
Group 7 (*n* = 9)	C57BL/6	ChABC	T8	BMS/grid	Anterograde BDA

*n.p.* not performed, *ChABC* chondroitinase, *Pase* penicillinase, *T8* thoracic level 8, *BMS* Basso Mouse Scale score, *grid* horizontal grid test, *DY* diamidine yellow, *BDA* biotinylated dextran amine

### Surgical procedures

Mice were anaesthetized using a combination of Hypnorm (fentanyl-citrate 0.7 mg/kg, fluanisone 22.5 mg/kg, Janssen Pharmaceutics) and Dormicum (midazolam 22.5 mg/kg, Roche Pharmaceuticals) administered via intraperitoneal injection.

Groups 4–7 underwent cortical injection of Pase or ChABC prior to SCI (details in Table 1). Mice were secured in a stereotaxic frame. A midline incision of the skin was performed and the location of the sensorimotor hindlimb cortex (smHL) was determined relative to Bregma. 1 μl of enzymatic solution was injected using a 33-gauge Hamilton syringe (1.25 mediolateral; −0.96 rostrocaudal; 0.6 dorsoventral). After injection, the needle remained in position for 2 min before removal to prevent spillage of the enzymatic solution.

In groups 2, 6 and 7, a dorsal bilateral laminectomy was performed at thoracic level 8 (T8) to expose the dura, and a bilateral dorsal spinal cord hemisection was performed using sharp iridectomy scissors. In 3 mice of group 2, a retrograde tracer was applied to the injury site as described below.

Following surgery, all animals were kept on a heating plate (36 °C) until fully awake. An analgesic (5 mg/kg body weight per subcutaneous injection of Rimadyl; Pfizer) and an antibiotic (5 mg/kg body weight intraperitoneal injection of Baytril; Bayer) were administered once per day for 3 days. Bladders were checked and emptied three times per day until their function had completely recovered.

### Tracing of the CST

Retrograde tracing (mapping of the axotomized smHL area): at the time of axonal injury, 1 μl of the retrograde tracer Diamidine Yellow (DY, 1 %; suspension in phosphate buffer (PB) 0.1 M and 2 % dimethyl sulphoxide, EMS-Polyloy, Gross-Umstadt, Germany) was applied at the lesion site with a 33-gauge Hamilton syringe mounted on the stereotaxic frame in 3 mice of group 2. The suspension was left on the transected surface for 15 min.

Anterograde tracing: 4 weeks after surgery, the CST of mice of groups 4–7 was anterogradely traced from the smHL ipsilateral to the treated cortex. Mice were anaesthetized as indicated above and secured in a stereotaxic frame. A midline incision of the skin was performed and the location of the smHL was determined by measuring positions on the skull relative to Bregma. 1-μl of biotinylated dextran amine (BDA) solution (10,000 MW, 10 % in PB 0.1 M, Molecular Probes) was injected in the smHL cortical area using a 33-gauge Hamilton syringe.

### Enzymatic treatment

To digest cortical CSPG GAG chains, protease-free chondroitinase ABC (ChABC) from *Proteus vulgaris* (Seikagaku) was reconstituted in sterile PB 0.1 M (0.1 U/μl, pH 7.4) and 1 μl was injected in the hindlimb area as indicated above. ChABC digests the GAG chains from the protein core of CSPGs that are diffusely expressed in the ECM or aggregated as PNNs. Pase (matched for protein content in PB 0.1 M, Sigma) was used as a control enzyme. To assess the area of enzymatic digestion, 4 mice per time point were euthanized at 2, 7 and 14 days after injection of ChABC, and brain coronal sections were stained with the lectin *Wisteria floribunda Agglutinin* (WFA) to visualize spared PNNs (see below for detailed staining protocol).

### Motor tests

For evaluation of over-ground locomotion and recovery of motor functions the Basso Mouse Scale (BMS) score was used (Basso et al. 2006). Mice were evaluated at day −1 and 3, 7, 14, 21 and 28 dpi. Mice were observed while walking in an open field (60 × 43 cm) by an experimenter blinded to the treatment groups and a score from 0 to 9 was assigned based on the following parameters: ankle movement, plantar placing, weight support, stepping, coordination, paw position, trunk stability. Prior to injury, groups 4–7 were trained for 3 days to walk on a horizontal grid. The horizontal grid (58 cm long × 20 cm width, 35 cm elevation) consisted of equally spaced rungs (at 1.2 cm intervals). Each mouse was allowed to walk on the grid for 3 min for 3 consecutive times, resting 25 min between each trial. On the third training day (−1 day), the three trials were video recorded to establish a baseline. Mice were further tested and videotaped at 3, 7, 14, 21 and 28 dpi. Videos were analysed offline. When the paw was placed such that the limb did not slip from the rung (correct weight-supported steps), a step was noted as successful. The percentage of footfalls was measured as the number of errors in foot placement out of the total number of steps during the 3-min recording or till a maximum of 50 steps per side. The percentage for each animal was determined by averaging over the first two trials. The right and left paws were analysed separately.

### Tissue processing

Mice were euthanized with an overdose of pentobarbital and transcardially perfused with Ringer (Fresenius Kabi, DE) supplemented with NaNO_2_ (40 mM), NaCHO_3_ (2 mM) and heparin (50 IE/ml, B. Braun Medical AG), followed by ice-cold 4 % paraformaldehyde in PB 0.1 M. The brains and spinal cords were dissected and post-fixed overnight at 4 °C in the same fixative. Brains were cryoprotected in a solution of 30 % sucrose in PB 0.1 M. Coronal sections (40 μm) were cut with a freezing microtome and serially collected as free-floating sections in PB 0.1 M. For PV and PNN staining, a complete series of sections (i.e. 12 sections taken at 240 μm intervals, spanning ~2.5 mm of tissue) was permeabilized and blocked in PB 0.1 M supplemented with 0.4 % triton-X100 (PB-TX 0.1 M) and 10 % inactivated normal horse serum for 2 h at room temperature. Sections were incubated with primary antibody/lectin (biotinylated *Wisteria floribunda* agglutinin (WFA), which labels the GAG chains of CSPGs in the ECM and PNNs, 1:500, B-1355 Vector Lab; mouse monoclonal anti-calcium-binding protein parvalbumin, 1:5000, PV235 Swant) at 4 °C for 3 days. After several washes in PB 0.1 M, slices were incubated with Streptavidin (Streptavidin AlexaFluor-555, 4 μg/ml, Invitrogen) or species-matched secondary antibodies (anti-mouse AlexaFluor-647, 4 μg/ml, Invitrogen) for 2 h at room temperature. Sections were counterstained with Dapi (1:10,000, 5 min, D3571 Invitrogen), washed in PB 0.1 M, and mounted onto gelatin-coated slides in Vectashield-mounting medium (Reactolab) to preserve fluorescent labelling. All immunostainings were performed in parallel on the same series of sections to minimize variability between groups.

Spinal cords were cleaned from their meninges before being divided into four parts corresponding to segments C1–C2, segments C3–C8, segments L1–L5, and a 4-mm piece containing the site of injury. The tissue was embedded in a gelatin-chicken albumin solution polymerized with 2.5 % glutaraldehyde as previously described (Herzog and Brosamle 1997). The segments C1–C2 were cut in the coronal plane (50 μm) to quantify the number of BDA-labelled CST fibres; horizontal sections (50 μm) of segments C3–C8 were used to quantify the number of CST collaterals; coronal sections (50 μm) of the lumbar spinal cords were used to assess completeness of the CST lesion (i.e. absence of BDA-stained CST axons) in injured mice; finally, cross-sections (50 μm) were examined at the site of injury to measure the size and depth of the lesion. All sections were collected in PB 0.1 M, serially mounted onto superfrost slides (SuperFrostPlus, Menzel-Glaser, Germany) and processed using the semifree-floating technique (Herzog and Brosamle 1997). In brief, sections were washed in TBS-0.3 % Triton X-100 (TBS-TX, 50 mM Tris, 0.875 % NaCl, 0.3 % Triton X-100, pH 8) before being incubated with avidin–peroxidase in TBS-TX (ABC elite, Vector Laboratories, Burlingame, CA, USA) for 1 h at room temperature. After an additional washing step in TBS-TX, a pre-incubation of 10 min in TBS-TX with 0.4 % ammonium nickel sulphate (Sigma, St. Louis, MO, USA) was performed, followed by a second pre-incubation of 10 min in TBS-TX with 0.4 % ammonium nickel sulphate and 0.015 % 3,3-diaminobenzidine (DAB; Sigma, Buchs, Switzerland). The peroxidase reaction was started by addition of 0.004 % H_2_O_2_ and was stopped after 30 min by extensive washes in PB 0.1 M. Sections were air-dried and coverslipped with Eukitt (Kindler, Freiburg, Germany). Sections containing the injury site were counterstained with cresyl violet to facilitate lesion size measurement. Incompletely injured or overhemisected mice were eliminated from all the analyses.

### Imaging and data analyses

Images of brain sections were acquired using a Leica TCS SP5 confocal inverted microscope or a Leica TCS SPE II confocal microscope (Leica Microsystems). Coordinates of the smHL cortex were defined by outlining the area including layer V pyramidal cell somata retrogradely labelled by DY injection at spinal cord level T8 (Neurolucida, MBF Biosciences). Mapping of GABAergic markers and PNNs was performed on 12 equally spaced (240 μm interval) sections per animal covering the entire smHL as well as rostral and lateral regions, i.e. caudal motor forelimb (mFL) cortex and lateral sensory forelimb (sFL) cortex, respectively (rostrocaudal from Bregma: +1.32; −1.16). Three sampling areas, extending from the pia to the white matter, were defined on the left hemisphere on each coronal section with a mediolateral extent of 500 μm, positioned at 250 μm intervals, starting 750 μm lateral to the midline. Mediolateral coordinates for sampling area 1 (0.75; 1.2); sampling area 2 (1.45; 1.95); sampling area 3 (2.2; 2.45). To count the number of cells positive for GAD67-GFP, PV and WFA, the sampling areas were subdivided in three regions of interest (ROI) according to the cortical layers identified by DAPI nuclear counterstaining (layers I–III, layer IV and layers V–VI). Cell counting was performed manually with Neurolucida software (MBF Biosciences) at a final magnification of 40×. All data are given as number of cells per ROI at the following coordinates: +1.08, +0.48, 0 (Bregma), −0.48 and −0.72. Sampling areas 1 and 2 allowed quantifying cells in the smHL and mFL, while sampling area 3 allowed quantifying cells in the sFL. Quantification of the expression level of GAD67-GFP, PV and WFA was performed in layer V of the smHL and mFL, where most of the changes in the number of cells expressing these markers were observed (see “Results”). Stacks were acquired in layer V at a final magnification of 40×. All acquisition parameters for the same marker were identical between groups. To insure randomized, unbiased selection of the analysed cells, a regularly spaced grid was superimposed on every picture and all cells lying under the axes of the grid were analysed. The optical density (OD) and the body area were measured by outlining the cell body perimeter of every single cell using the LAS AF software (Leica microsystems, Mannheim, Germany). To minimize possible variability coming from the staining protocol, all values were normalized for the average intensity obtained from the control group. Collaterals in C3–C8 of intact and injured spinal cords were counted in complete series of adjacent, horizontal sections using the Neurolucida software (MBF Biosciences). The absolute number of collaterals was divided by the length of the analysed CST. To normalize for variation in tracing efficiency, these numbers were further divided by the total number of traced CST fibres counted at cervical level C1–C2 (average of 3 counts). The resulting index represents the number of cervical collateral per millimetre per labelled smHL CST fibres. The size of the SCI was estimated for all mice of groups 2, 6 and 7 by measuring the area of spared spinal cord tissue at the lesion epicentre.

All analyses were performed on a blinded basis. All values in the figures and in the text are presented as the mean ± standard error of the mean. Statistical analyses were performed using two-way ANOVAs with the Bonferroni post hoc test or the Student’s *t* test.

## Results

### Mapping of the axotomized corticospinal neurons in the sensorimotor hindlimb cortex

**Fig. 1:**
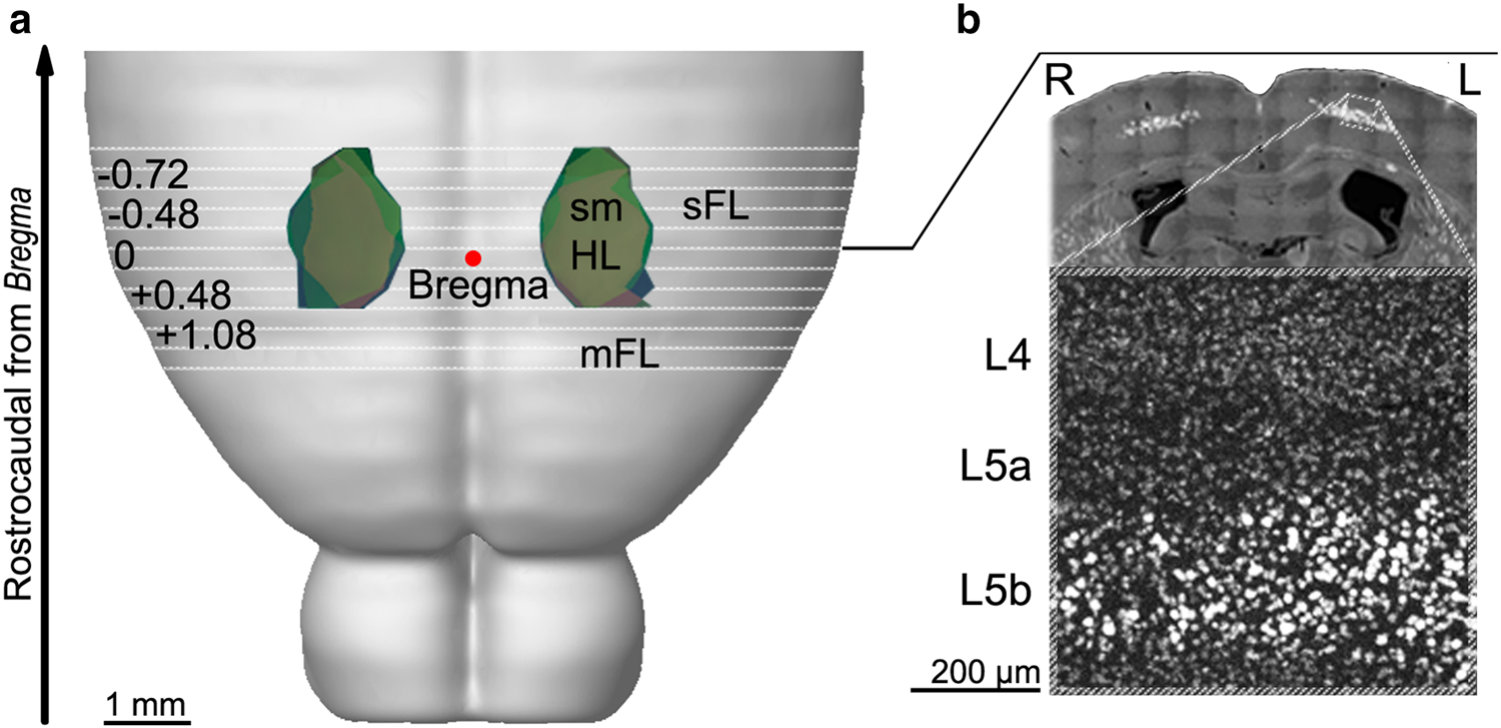
Mapping of axotomized hindlimb-projecting corticospinal neurons in the mouse sensorimotor hindlimb (smHL) cortex. **a** Axotomized corticospinal neurons were retrogradely labelled by injection of the tracer Diamidine Yellow (DY) at the site of spinal cord injury (i.e. T8). Serial coronal sections of the forebrains were used to map the distribution of labelled cell bodies. The resulting areas are identified in three mice (*blue*, *green* and *pink areas*) and superimposed onto a brain template (http://wholebraincatalog.org). The rostrocaudal (+0.6 mm; −1.4 mm) and mediolateral (largest extension: 0.76 to 1.78 mm) coordinates of the smHL are shown in reference to Bregma (*red spot*) and were consistent between mice. *White lines* represent the serial sections used for the analyses. *mFL* motor forelimb, *sFL* sensory forelimb. **b** Dapi staining (*dark grey*) was used to visualize the cortical layers. DY+ cell bodies of axotomized pyramidal neurons (*light grey*) were located in layer 5b. *R* right, *L* left, *L4* layer 4, *L5a* layer 5a, *L5b* layer 5b

To accurately map the region of the mouse sensorimotor cortex innervating the lumbar spinal cord, we retrogradely labelled the axotomized corticospinal neurons by injecting the tracer Diamidine Yellow (DY) at the site of injury (i.e. T8 dorsal hemisection). Serial coronal sections of the mouse forebrains were used to map the distribution of labelled cells and to define the coordinates of the sensorimotor hindlimb (smHL) cortex. The rostrocaudal (+ 0.6 mm; −1.4 mm) and mediolateral (largest extension 0.76–1.78 mm) coordinates were set with reference to Bregma and were consistent between mice (Fig. 1a). All axotomized pyramidal neuron somata were found in layer 5b (Fig. 1b). The regions located immediately rostral and lateral to the smHL cortex were defined as motor forelimb (mFL) and sensory forelimb (sFL) areas, respectively (Ayling et al. 2009; Brown et al. 2009; Ghosh et al. 2012). The defined coordinates were used to select the sampling areas in which quantifications of GABAergic markers and PNNs were done.

### Complex spatial changes of GABAergic markers occur in the sensory and motor cortex after SCI

**Fig. 2:**
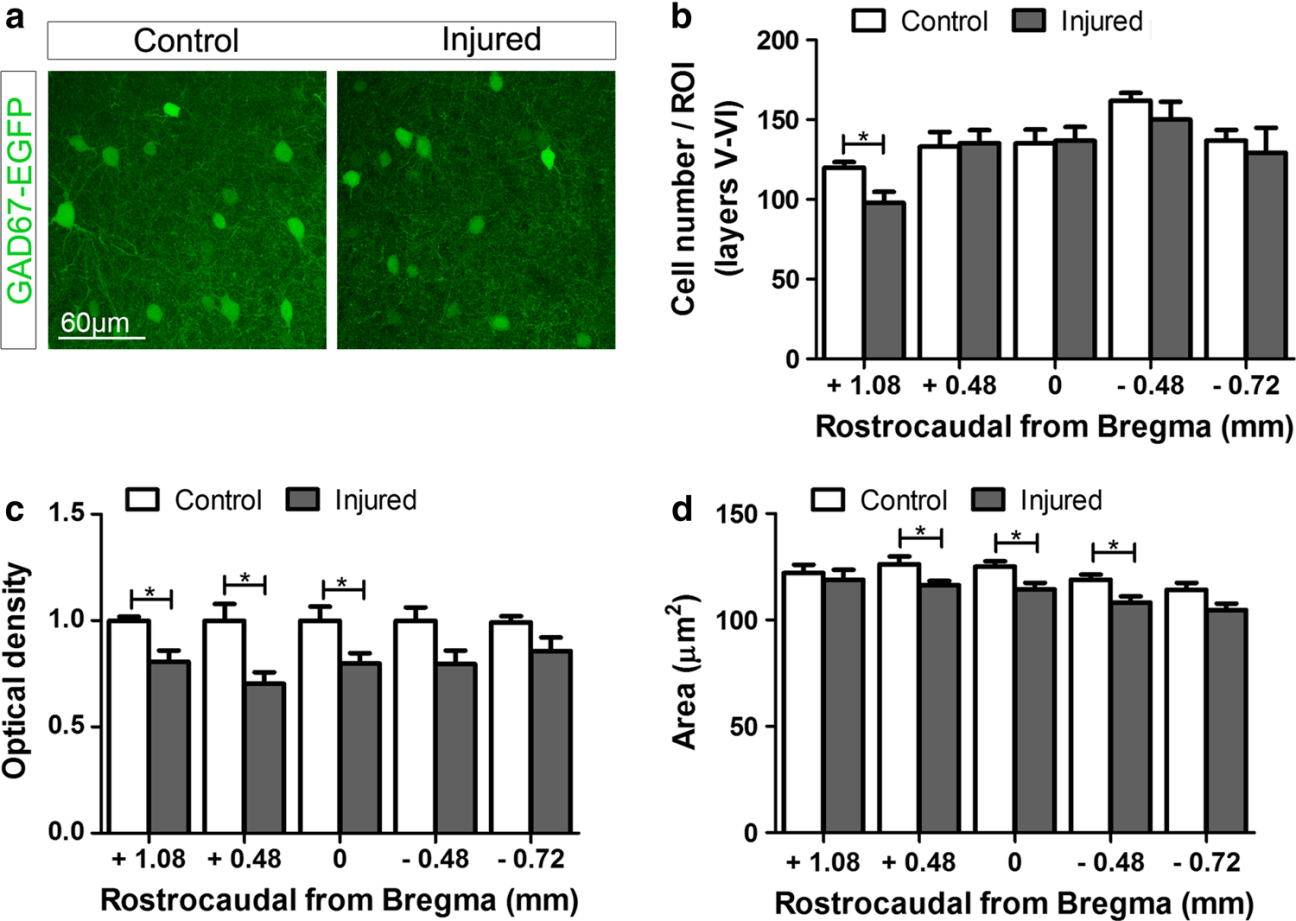
Analysis of GAD67-GFP+ cells in cortical layers V–VI of control and injured mice. **a** Representative images showing GAD67-GFP+ cells in layer V of control (*left*) and injured (*right*) mice at +0.48 mm from Bregma. **b** The total number of GAD67-GFP+ cells was significantly decreased in the mFL of injured (*grey bars*) compared to control (*white bars*) mice. No differences were observed in the smHL cortex of the two groups. **c** Optical density measurement of single cells in layer V of the smHL and mFL cortex revealed that the expression of GAD67-GFP was significantly lower in injured (*grey bars*) compared to control (*white bars*) mice at the boundary between these two areas. **d** Measurement of GAD67-GFP+ cell body area revealed a small but significant atrophy of this cell population exclusively in the smHL cortex of injured (*grey bars*) compared to control (*white bars*) mice. No atrophy could be observed in the mFL cortex. *p < 0.05. Student’s *t* test

We next studied the effects of a thoracic SCI on the spatial distribution of GAD67-GFP+, PV+ and WFA-labelled PNNs (WFA+ PNNs) throughout all cortical layers of the mFL, smHL and sFL. In addition, we focused on layer V of the mFL and smHL cortical areas and measured the staining intensity (optical density of single cells) of the three markers and the cell body area of GAD67-GFP+ and PV+ cells (group 1, control, *n* = 4; group 2, T8 injured, *n* = 7). No changes could be detected in the total number of GAD67-GFP+ cells in any of the cortical layers of the smHL cortex (Fig. 2b and Online Resource 1), suggesting that no GABAergic cell death had occurred in the region where axotomized neurons were located. Interestingly however, the number of GFP+ interneurons in layers I–III and V–VI was significantly decreased in the mFL area (+1.08 rostrocaudal; 1.37 mediolateral; layers I–III, *p* = 0.027, Online Resource 1; layers V–VI, *p* = 0.04, Fig. 2b) and increased in the caudal sFL area (−0.72 rostrocaudal; 2.5 mediolateral; layers I–III, *p* = 0.037; layers V–VI, *p* = 0.011, Online Resource 1). These changes are unlikely to be related to death or birth of new GAD67+ cells, but rather suggest modulation of GAD67 expression after SCI. Regional changes in GAD67-GFP expression levels were confirmed by single cell body optical density measurement in layer V of the rostral smHL and mFL. In this regions, the intensity level of GAD67-GFP was significantly lower in injured compared to control mice (+1.08 *p* = 0.024; +0.48 *p* = 0.01; 0 *p* = 0.036, Fig. 2a, c). In the mFL cortex, this decrease could largely be attributed to GABAergic PV-negative (GAD67-GFP+/PV−) cells, i.e. calretinin and/or somatostatin cells (Tamamaki et al. 2003), (Online Resource 2). In the smHL cortex, GABAergic interneurons that had downregulated PV following injury (see below) but could still be identified by their PNNs (GAD67-GFP+/WFA+/PV−) also contributed to this decrease (Online Resource 2). Finally, measurement of GAD67-GFP+ cell body area revealed a small but significant atrophy of this cell population exclusively in the smHL area, where axotomized CST neurons were located (+0.48 *p* = 0.027; 0 *p* = 0.047; −0.48 *p* = 0.033, Fig. 2d). No atrophy could be observed in the mFL area indicating that the lower level of GAD67-GFP expression did not influence cell body area measurements.

**Fig. 3:**
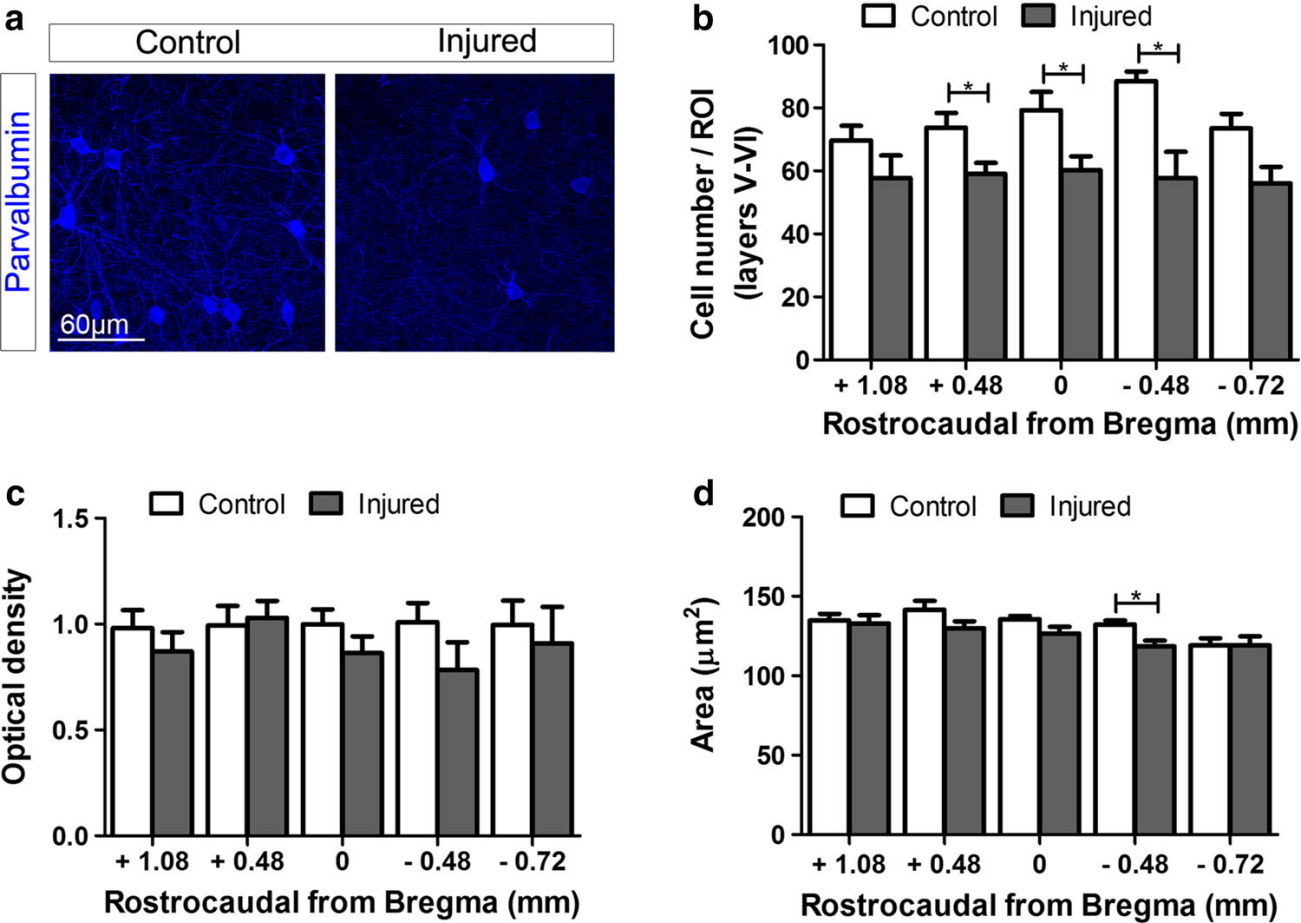
Analysis of PV+ cells in cortical layers V–VI of control and injured mice. **a** Representative images showing PV+ cells in layer V of control (*left*) and injured (*right*) mice at +0.48 mm from Bregma. **b** The total number of PV+ cells was significantly decreased throughout the deeper layers (layers V–VI) of the smHL cortex of injured (*grey bars*) compared to control (*white bars*) mice. No differences were observed in the mFL cortex of the two groups. **c** Optical density measurement of single cells in layers V of the smHL and mFL cortex revealed that the expression levels of PV were comparable in the positive cell populations of control (*white bars*) and injured (*grey bars*) mice. **d** Atrophy of PV+ cells was minimal in injured mice (*grey bars*) and only reached statistical difference at −0.48 mm caudal from Bregma compared to the control group (*white bars*). *p < 0.05. Student’s *t* test

We next focused our analysis on PV+ interneurons, which represent the primary source of cortical lateral inhibition on pyramidal neurons (Freund and Katona 2007). In the rodent cortex, PV+ cells are present in all layers except layer I and reach their peak density in the middle layers (van Brederode et al. 1991). Virtually all PV+ interneurons in the cortex also express GAD67 (Tamamaki et al. 2003). A significant decrease in the number of PV+ cells was found throughout layers V–VI of the smHL cortex (+0.48 *p* = 0.033; 0 *p* = 0.027; −0.48 *p* = 0.024, Fig. 3a, b). These changes were largely specific to the deeper layers, as no significant decrease was observed in more superficial layers of the same area or in other areas (Online Resource 3) and suggested a downregulation of the protein exclusively in a subpopulation of PV+ cells. Indeed, measurements of single cell body optical density revealed that PV expression level remained stable in the population of detectable cells (Fig. 3a, c). Atrophy of PV+ cells was minimal and only reached statistical difference at −0.48 mm caudal to Bregma (*p* = 0.03, Fig. 3d). These data identify the presence of two subpopulations of PV cells in the deeper layers of the smHL cortex which differently responded to the injury: one where PV expression (and to some extent GAD67-GFP, see above) is persistently downregulated (cell undetectable by PV immunostaining) compared to control mice, and one that remains largely unaffected (cells detected by PV immunostaining).

Taken together these data indicate that SCI results in complex topographic and layer-specific changes in the expression of GABAergic markers in sensory and motor cortical areas. Moreover, they reveal that atrophy of inhibitory interneurons occurs in cortical layers where axotomized CST neurons are located.

### SCI influences integrity of cortical PNNs at the boundary between mFL and smHL

**Fig. 4:**
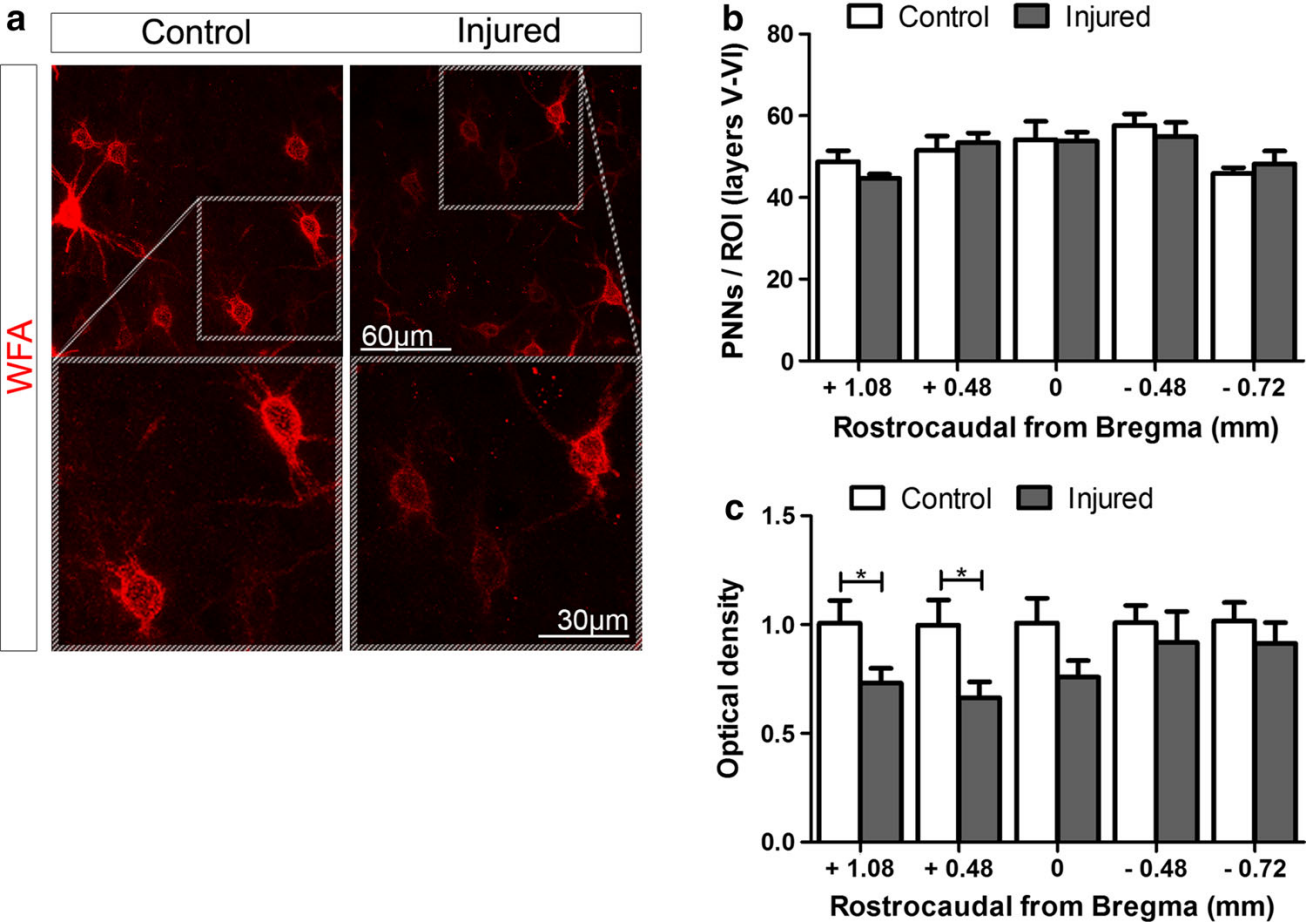
Analysis of PNN surrounded cells in cortical layers V–VI of control and injured mice. **a** Representative images showing WFA+ PNNs in layer V of control (*left*) and injured (*right*) mice at +0.48 mm from Bregma. In this region, PNNs of injured mice appeared thinner and fainter compared to control mice. **b** No changes in the total number of WFA+ PNNs were observed in injured (*grey bars*) compared to control (*white bars*). **c** Optical density measurement of single nets revealed a reduced WFA staining intensity at the boundary between mFL and smHL cortical areas. *p < 0.05. Student’s *t* test

To test for changes in CSPG GAG chain integrity, we focused our analysis on WFA+ PNNs (CSPG aggregates), which surround cortical PV cells. No changes in the total number of PNNs were observed in injured compared to control mice in any of the cortical areas and layers analysed (Fig. 4b and Online Resource 4). However, optical density measurement of single nets revealed a reduced WFA intensity staining at the boundary between mFL and smHL cortical areas (+1.08 *p* = 0.046; +0.48 *p* = 0.03, Fig. 4a, c). In this region, PNNs appeared thinner and fainter in injured compared to control mice (Fig. 4a). To gain insight into the interplay between PV and integrity of PNNs, we measured the optical density of WFA+ PNNs in individual PV+ (GAD67-GFP+/WFA+/PV+) and PV− (GAD67-GFP+/WFA+/PV−) cells. This analysis revealed that a reduction of WFA intensity occurred in both PV+ and PV− subgroups suggesting that changes in cortical integrity of PNNs and PV expression after SCI are not directly related (Online Resource 5).

It is interesting to note that the reduction of PNN staining intensity occurred in a cortical region (at the boundary between the more rostral smHL and caudal mFL cortex) where axotomized CST neurons undergoing spontaneous anatomical and functional remodelling after SCI are located (Bareyre et al. 2004; Fouad et al. 2001; Ghosh et al. 2010). In particular, axotomized CST neurons located in the rostral smHL cortex form cervical axonal collaterals, which support recovery of motor functions after thoracic SCI (Fouad et al. 2001; Ghosh et al. 2010). In light of evidence showing restriction of structural and functional plasticity mediated by PNNs in the mature CNS, we hypothesized that changes in cortical PNN CSPGs integrity after SCI might be instrumental for CST remodelling in the spinal cord. We therefore tested if experimental widespread digestion of cortical CSPG GAG chains would have influenced spontaneous anatomical CST plasticity and recovery of motor functions.

**Fig. 5:**
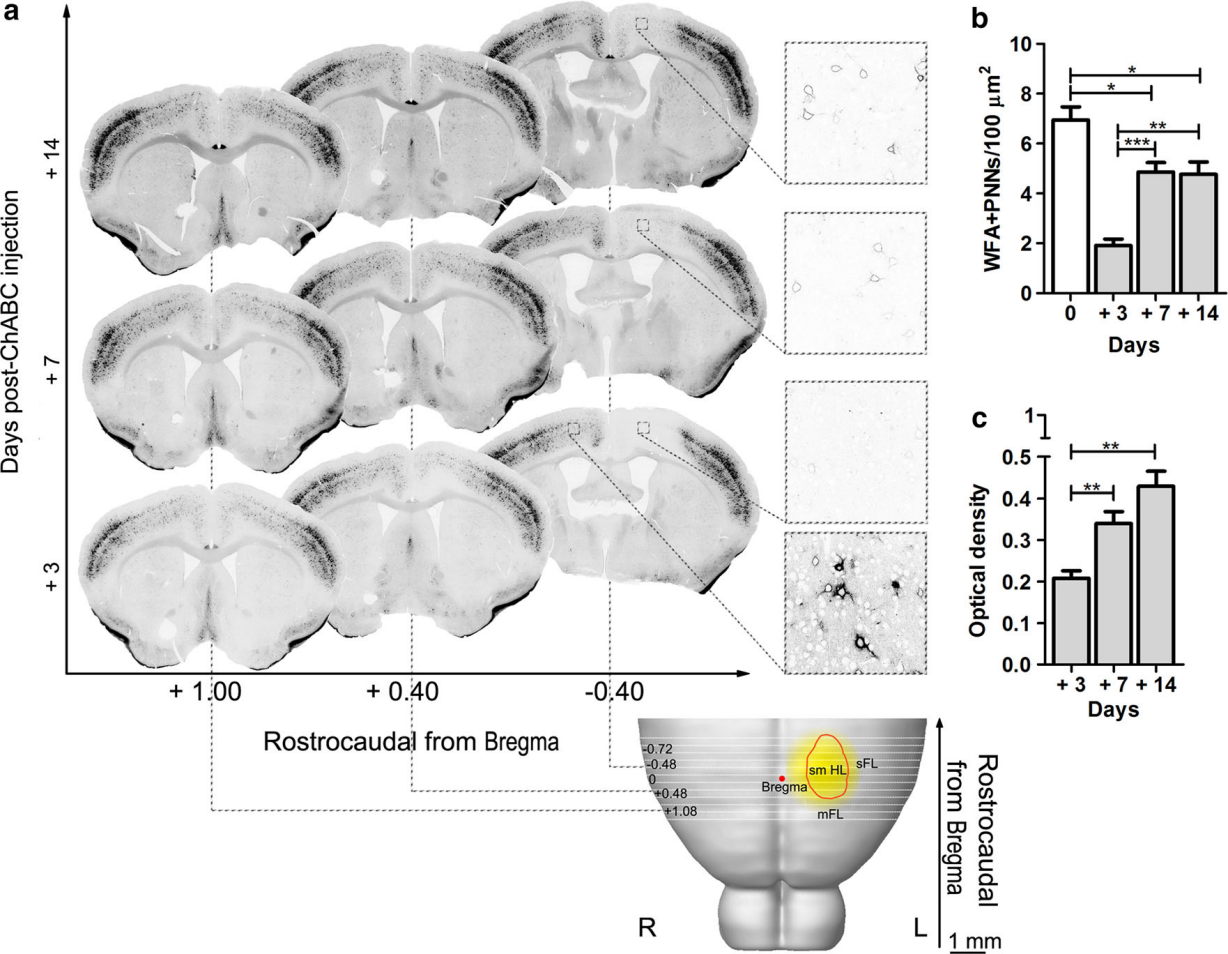
Reconstruction of ChABC-digested area and gradual reappearance of PNNs at 3, 7 and 14 days after ChABC injection. **a** The digested area (*yellow*) was superimposed onto a brain template (http://wholebraincatalog.org). The *orange circle* outlines the smHL area. The area devoid of WFA staining had a rostrocaudal extension of 2.709 ± 0.125 mm and included the boundary between mFL and smHL, and the smHL cortex. Magnifications show PNNs in the digested area (ipsilateral to the injection) and in the contralateral vehicle-treated hemicortex (only at 3 days post-injection). Note the gradual reappearance of PNNs at 7 and 14 days as thin and faint structures. **b**, **c** In the digested area, the density of WFA+ PNNs was below control levels (day 0) at all time points analysed and a gradual increase of WFA staining intensity could be observed. *p < 0.05, **p < 0.01, ***p < 0.001. *R* right, *L* left. Student’s *t* test

We injected ChABC or the control enzyme Pase into the left smHL cortex of C57/Bl6 mice in combination with (groups 6 and 7) or without a complete T8 CST transection (groups 3–5). At 3 days post-injection, the area of enzymatic digestion had a rostrocaudal extension of 2.709 ± 0.125 mm starting at +0.942 ± 0.137 mm from Bregma, thus including the boundary between mFL and smHL and the smHL cortex. This area was almost completely devoid of PNNs (Fig. 5a). In the digested area, measurement of WFA-stained sections at 3, 7 and 14 days after ChABC treatment revealed a gradual reappearance of PNNs (Fig. 5a–c). The density of WFA+ PNNs however remained below control levels at all time points (number of WFA+ PNNs/100 μm^2^ at day 3 after the injection: 1.91 ± 0.26, *p* < 0.001; 7: 4.86 ± 0.37, *p* = 0.03; 14: 4.77 ± 0.49, *p* = 0.03, Fig. 5b), and was significantly different 3 days after injection compared to both longer time points (3 *vs.* 7 days, *p* < 0.001; 3 *vs.* 14 days, *p* < 0.01).

### Intracortical ChABC injection transiently exacerbates motor deficits

**Fig. 6:**
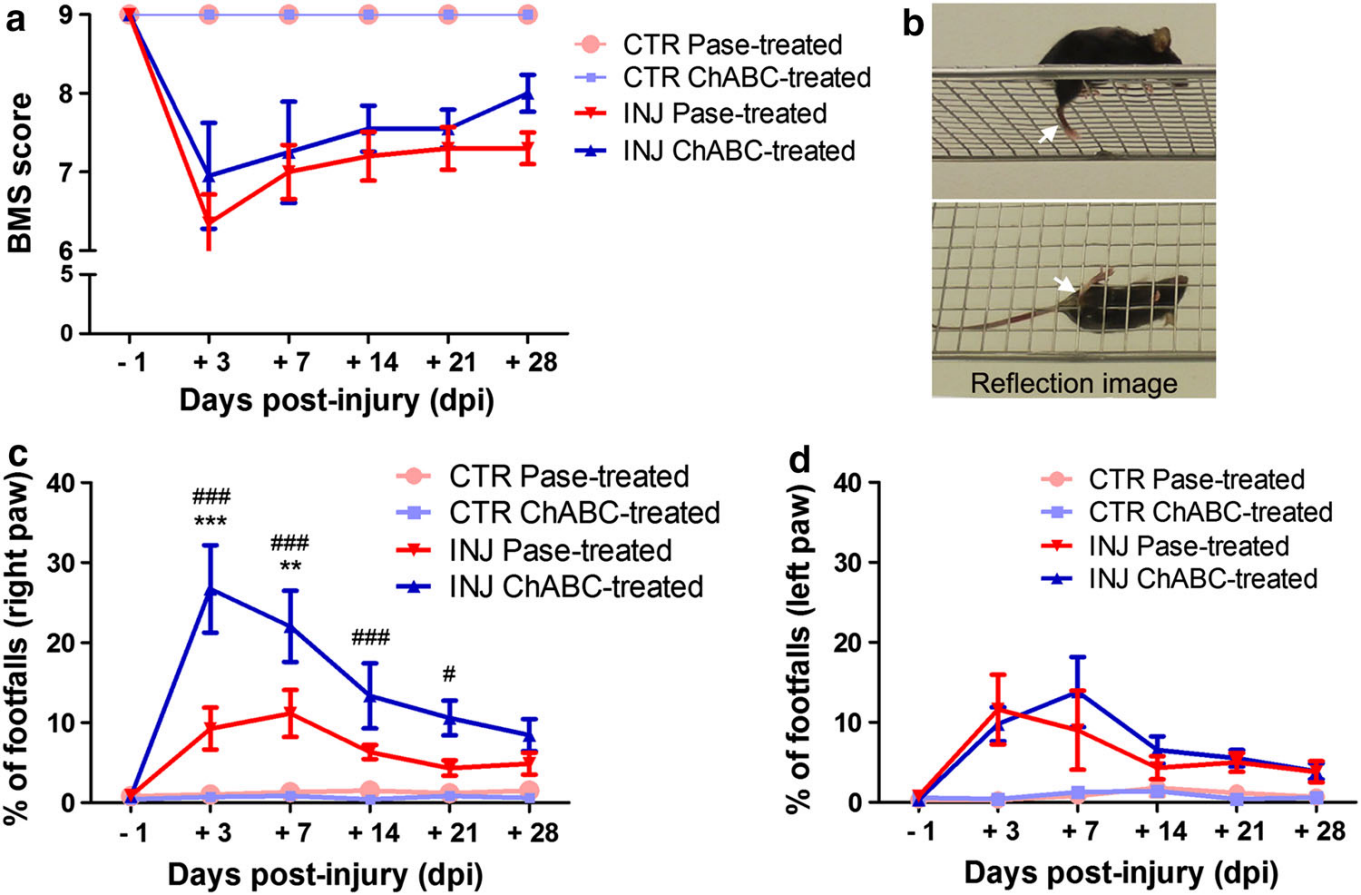
Intracortical ChABC injection transiently exacerbates motor deficits. **a** Functional recovery of over-ground locomotion was assessed with the Basso Mouse Scale (BMS) score before (−1 days) and after injury (+3, 7, 14, 21, 28 days). The two control unlesioned groups (groups 4, *pink*; group 5, *light blue*) reached the highest score throughout the testing period. The locomotor rating scale of the two injured groups (group 6, *red*; group 7, *blue triangles*) was always significantly lower than the control groups. No differences were detected between the scores of the injured groups at any time point. **b** Representative snapshot of a video recording showing a footfall (hindpaw slips) from the grid. The lower image is given by the reflection in a mirror positioned below the grid. *White arrows* indicate the slipping hindpaw. **c** Mice of both injured groups 6 (group 6, *red*) and 7 (group 7, *blue*) showed poorer performances in the first 2 weeks after injury compared to the respective control mice (groups 4, *pink*; group 5, *light blue*). Stepping with the right hindpaw (opposite to the site of cortical injection) was more severely impaired in mice treated with ChABC (group 7, *blue*), when compared to injured Pase-treated mice (group 6, *red*). The severity of this deficit reached the highest and significant levels at 3 and 7 dpi, when cortical PNNs were strongly digested (see Fig. 5a–c), and returned to values comparable to injured Pase-treated mice at 14 dpi, when PNNs reappeared in the digested area. **d** The time course of spontaneous recovery for the left hindpaw of injured ChABC-treated mice (group 7, *blue*) was comparable with the left and right hindlimbs of injured Pase-treated mice (group 6, *red*). Group 7 *vs.* group 6: **p < 0.01, ***p < 0.001; group 7 *vs.* group 5: ^#^ *p* < 0.05, ^###^ *p* < 0.001, two-way ANOVA with Bonferroni post hoc test

To address the effects of cortical CSPG GAG chains digestion on motor function recovery, mice of groups 4–7 were assessed using the Basso Mouse Scale (BMS) score and the horizontal grid walking test at different time points after injury. The BMS score evaluates over-ground locomotor activity in an open field. The two control unlesioned groups (groups 4 and 5) reached the highest score throughout the testing period (Fig. 6a) indicating that neither ChABC nor Pase intracortical injection per se affected the gross locomotor ability of uninjured mice. During the 4 weeks post-injury, the locomotor rating scale of the injured groups 6 and 7 remained significantly lower than the control groups (group 4 and 5, respectively) and never recovered to control level. Importantly, the BMS scores were consistent between injured mice and comparable between the two groups, suggesting that locomotion and the extent of the lesion were similar in all mice.

The horizontal grid test examines the deficits in descending fine motor control and therefore represents a more specific test to assess CST integrity. Video recording allowed us to quantify the number of hindpaw slips (footfalls, Fig. 6b) on both sides (i.e. contralateral and ipsilateral to the injection). Digestion of CSPGs in the sensorimotor cortex of unlesioned mice did not affect CST-dependent descending motor control (Fig. 6c, d). All unlesioned mice of groups 4 and 5 (Pase and ChABC injected, respectively) walked on the grid with ease, showing correct hindpaw placement. In contrast, mice of both injured groups 6 and 7 showed poor performance in the first 2 weeks after injury when compared with the respective control mice (Fig. 6c, d). Interestingly, stepping with the right hindpaw (opposite to the site of cortical injection) was more severely impaired in injured mice treated with ChABC (i.e. group 7), when compared with injured Pase-treated mice (i.e. group 6). The severity of this deficit reached the highest and significant levels at 3 and 7 dpi (at 3 dpi ***p < 0.001; at 7 dpi **p < 0.01 two-way ANOVA with Bonferroni post hoc test), when cortical PNNs were fully digested, and returned to values comparable to injured Pase-treated mice at 14 dpi, when PNNs-CSPGs reappeared in the digested area (Figs. 5a, 6c). By 28 dpi, both injured groups had recovered fine motor function of the right hindpaw to control levels.

All together these data indicate that cortical CSPG GAG chains digestion transiently exacerbates functional CST-associated motor deficits after thoracic SCI.

### Intracortical ChABC injection impairs the spontaneous intraspinal sprouting of axotomized CST neurons

**Fig. 7:**
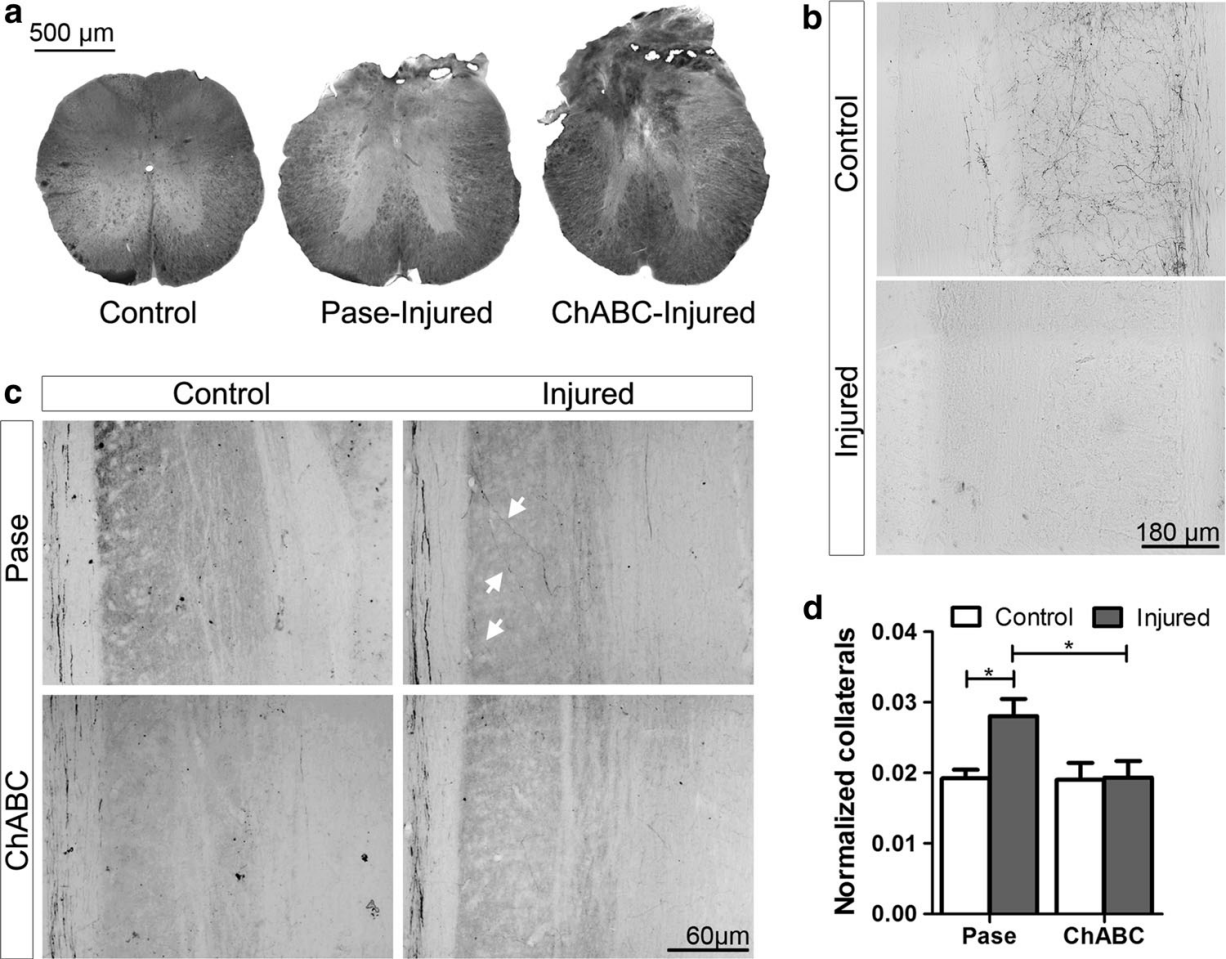
Intracortical ChABC injection impairs spontaneous long-term intraspinal sprouting of axotomized CST neurons. **a** Representative images showing the area of spared spinal cord tissue in control mice (control) and at the centre of the lesion in injured Pase-treated (group 6) and injured ChABC-treated mice (group 7). No significant difference was detected between groups. **b** Representative images showing the absence of BDA-positive fibres in the lumbar enlargement caudal to the lesion in injured ChABC-treated (*lower picture*) compared to control ChABC-treated (*upper picture*) mice. **c** Representative images showing BDA+ collaterals in horizontal sections of the cervical (C3–C8) spinal cord of mice in groups 4 to 7. White arrows indicate collaterals in Pase-treated mice (group 6). **d** The number of collaterals from CST axons was significantly increased in injured Pase-treated mice compared to control unlesioned mice of both groups. In group 7 (injured ChABC-treated mice) the number of cervical collaterals was significantly lower than in group 6 and not different from the two control unlesioned groups. *p < 0.05, Student’s *t* test

Sprouting of the CST has been shown to occur spontaneously after SCI, leading to the formation of new intraspinal functional circuits (Bareyre et al. 2004) that participate in the functional reorganization of the sensorimotor cortex (Fouad et al. 2001; Ghosh et al. 2009). Thus, immediately after completion of behavioural testing (28 dpi), the CST originating in the left hemisphere was anterogradely traced with BDA in groups 4–7, and the animals were euthanized 2 weeks later. The size of the SCI was estimated by measuring the area of spared spinal cord tissue at the centre of the lesion (Fig. 7a). No significant difference was detected between groups 6 and 7 (average spared spinal cord tissue in group 6: 0.88 ± 0.05 mm^2^, in group 7: 0.91 ± 0.06 mm^2^, *p* = 0.755; percentage compared to intact spinal cord in group 6: 59.52 %, in group 7: 61.16 %). In all mice of groups 6 and 7, the CST was completely transected as demonstrated by the absence of BDA-positive fibres in the lumbar enlargement caudal to the lesion (Fig. 7b). Large portions of the dorso-lateral and ventral funiculi were spared by the lesion (Fig. 7a). Cortical diffusion of BDA was comparable between all groups, with no tracer spreading into the deep white matter or subcortical structures. BDA+ cell bodies were found solely in the smHL cortex with no cells observed in the mFL cortex. No significant difference was observed in the number of BDA-labelled fibres in the four groups (cross-section at level C1–2) indicating that neither ChABC nor Pase injections impaired BDA uptake or induced a loss of CST neurons. Collaterals were analysed in horizontal sections of the cervical (C3–C8) spinal cord (Fig. 7c, d). The number of collaterals from CST axons was significantly increased in the injured Pase-treated mice compared to control unlesioned mice (unlesioned Pase-treated mice, group 4, *p* = 0.015; unlesioned ChABC-treated mice, group 5, *p* = 0.021). Surprisingly, in group 7, which received a SCI and ChABC cortical injection, the number of cervical collaterals was significantly lower than in group 6 (*p* = 0.02) and not different from the two control unlesioned groups, indicating that digestion of cortical CSPG GAG chains abolished spontaneous long-term sprouting of axotomized CST neurons rostral to the site of injury.

## Discussion

In this study, we showed that a thoracic SCI in mice is followed by specific spatial changes in the expression of GAD67 and PV in motor and sensory cortical areas, as well as by reduced WFA-stained PNNs at the boundary between the smHL and mFL cortical areas. Digestion of CSPG GAG chains over a larger sensorimotor cortical area by intracortical ChABC injection was used as a strategy to promote anatomical reorganization of the CST and motor function recovery. Our results however showed that intracortical ChABC injection prevented CST sprouting rostral to the site of SCI and worsened motor control deficits, suggesting that these cortical changes must be discrete and tightly controlled to be of functional benefit.

Our results highlighted that a number of spatially restricted changes occurred in GABAergic circuits of the sensorimotor cortex after SCI. We detected an atrophy of layer V interneurons throughout the smHL, but not in the adjacent mFL cortex. The smHL contains the cell bodies of axotomized CST neurons, which undergo severe atrophy (Nielson et al. 2010; Carter et al. 2008; Tang et al. 2004; McBride et al. 1989; Ghosh et al. 2012) as a consequence of loss of trophic support (Tetzlaff et al. 1994; Giehl and Tetzlaff 1996; Kobayashi et al. 1997). The decreased metabolic activity of the axotomized pyramidal neurons onto which GAD67+ cells project might have locally impaired trophic support and triggered the observed atrophy of interneurons. Such a dependence of interneurons to pyramidal cells integrity is supported by the observation of a similar pattern of GABAergic neurons atrophy after projection neuron apoptosis (Al-Abdulla and Martin 2002).

Systematic cell number quantification and intensity measurement also revealed a decreased expression of GAD67 and PV in the sensorimotor cortex after SCI. The decrease of these activity-dependent GABAergic markers (Lau and Murthy 2012; Kinney et al. 2006) strongly supports the prevailing view that cortical disinhibition underlies experience-driven circuits remodelling. It is likely that these cortical changes were triggered by the transection of ascending sensory afferences, as suggested by Ghosh et al. (2009) and studies in the visual cortex showing that eye removal, TTX injection or monocular deprivation leads to similar observations (Hendry and Jones 1988; Sale et al. 2007). Interestingly, a similar decline of cortical GAD67 and PV expression has also been reported in various CNS pathologies, such as psychiatric disorders (Reynolds et al. 2004), Alzheimer’s disease (Solodkin et al. 1996), epilepsy (Lau et al. 2000) as well as during cortical microstimulation-induced circuit remodelling (Benali et al. 2008). Under all these conditions, the decreased expression of those markers is believed to indicate a change of neuronal network activity. Interestingly, our work revealed that these changes occurred in discrete regions and/or layers of the sensorimotor cortex after SCI. Two weeks after SCI, downregulation of GAD67 expression was observed across the caudal mFL and rostral smHL cortex, whereas a reduced number of PV+ cells was only detected in the deeper layers of the axotomized smHL cortex. This complex pattern of disinhibition of cortical circuits might support the profound remodelling occurring in the mFL and smHL cortex after SCI in rodents (Fouad et al. 2001; Ghosh et al. 2010, 2012). Interestingly, opposite changes (i.e. increased GAD67 expression) were observed in the intact caudal sFL, possibly aimed at confining the reorganization of cortical circuits to precise cortical sensorimotor areas (Benali et al. 2008).

In parallel to the interneuron atrophy and changes in activity-dependent GABAergic marker expression reported above, we observed a spatially localized reduction in the integrity of CSPG GAG chains associated with PNNs (revealed as significant reduction in the intensity of WFA staining). This reduction was more pronounced at the transition region between the mFL and smHL cortical areas, a region containing pyramidal neurons that spontaneously sprout into the cervical spinal cord after thoracic SCI (Fouad et al. 2001; Ghosh et al. 2010). Accumulating evidence support the existence of an interplay between experience-dependent plasticity and integrity of PNNs. PNNs appear at the end of the critical period for plasticity and persist in adulthood where they restrict functional plasticity of mature neuronal circuits (Kwok et al. 2011). Knocking out components essential for their assembly prolongs a juvenile state of plasticity into adulthood (Carulli et al. 2010). Enhanced expression of metalloproteinases (MMPs), that can degrade CSPGs forming PNNs, promotes experience-dependent cortical reorganization (Wang et al. 2008; Michaluk et al. 2011), and is observed together with reduced density of PNNs in cortical areas undergoing reorganization after stroke (Bidmon et al. 1998; Hobohm et al. 2005). In light of these data, our results suggested that changes in the integrity of cortical CSPG GAG chains, particularly in PNNs, might contribute to the promotion of motor circuit remodelling after SCI. To test if the reduction of cortical PNN CSPG integrity observed in the rostral smHL was instrumental to compensatory plasticity of the CST, we performed unilateral ChABC injection to digest their GAG chains over a larger area of the smHL cortex. Instead of promoting recovery, our results indicated that broad digestion of cortical CSPG GAG chains, revealed as the absence of WFA-stained PNNs, was detrimental to compensatory CST plasticity after SCI. This contrast with previous studies reporting that intraspinal or intrathecal delivery of ChABC after SCI promotes anatomical and functional repair of both descending motor (Barritt et al. 2006; Bradbury et al. 2002; Carter et al. 2008; Garcia-Alias et al. 2008, 2009; Fouad et al. 2005) and ascending sensory projections (Cafferty et al. 2008). A key difference between these previous studies and our study is the site of ChABC delivery. Thus, while ChABC activity at or around the site of SCI promotes repair, intracortical injection of this enzyme (our study) results in transient aggravation of behavioural deficits. We indeed observed rapid (i.e. at 3 and 7 dpi) and limb-specific worsening of motor function recovery, which eventually disappeared at longer time points (i.e. 28 dpi) when cortical PNNs reappeared in the digestion area. The early occurrence of these deficits suggests that ChABC-mediated digestion of PNN CSPGs disrupted rapid changes taking place in the sensorimotor cortex after SCI, such as reduction of intracortical inhibition and unmasking or reorganization of synapses which sustain motor function recovery (Jacobs and Donoghue 1991; Levy et al. 2002; Aguilar et al. 2010). It is possible that, by perturbing these rapid changes, ChABC treatment delays the formation and/or consolidation of new meaningful cortical circuits following injury. Impairment of motor function recovery after intraspinal delivery of ChABC in an animal model of SCI was also reported by Garcia-Alias et al. (2009). In this study, ChABC treatment induces worsening of motor tasks that were not actively trained after the injury. Interestingly, ChABC has a persistence of at least 10 days after a single injection in vivo (Lin et al. 2008), which roughly correspond to the timing of worsening of motor recovery that we observed in ChABC-treated animals. Afterwards, the replacement of newly synthesized glycans in PNNs might parallel or initiate compensatory mechanisms in the cortex to support spontaneous motor function recovery at longer time points (21 and 28 dpi). The nature of these compensatory mechanisms is likely to be diverse, including changes not only of neuronal circuitries but also of neuronal intrinsic properties. For example, a similar transient phenotype was recently reported for pyramidal cells excitability after downregulation of GAD67 in PV+ interneurons in juvenile mice, illustrating the exquisite capacity of cortical circuits to adapt to pathological situations (Lazarus et al. 2013).

Intracortical ChABC injection also blocked the spontaneous sprouting of axotomized CST fibres in the cervical spinal cord (Bareyre et al. 2004; Fouad et al. 2001; Ghosh et al. 2009). Modulation of the physiological properties of PNNs-surrounded PV+ interneurons after ChABC treatment (Dityatev et al. 2007) might influence the early network remodelling induced by SCI and drive changes in cortical network activity, thereby influencing CST sprouting abilities (Salimi et al. 2008). However, changes of dendritic structural and functional plasticity mediated by diffused CSPGs cannot be ruled out (Orlando et al. 2012; de Vivo et al. 2013). CSPGs signalling through the protein tyrosine phosphatase sigma (Shen et al. 2009), the leukocyte common antigen-related phosphatase (Fisher et al. 2011) and the Nogo receptor family members NgR1 and NgR3 (Dickendesher et al. 2012) is known to affect plasticity. In addition, CSPGs may modulate signalling of growth promoting factors (e.g. Koprivica et al. 2005). Interestingly, despite the absence of CST sprouting in ChABC-treated mice, these animals eventually recovered to the level of the control Pase-treated group. This implies that a significant degree of functional recovery was achieved that did not involve CST sprouting in the cervical spinal cord. This spontaneous recovery most probably involves the reorganization of other parallel descending motor pathways (Raineteau and Schwab 2001).

Taken together our findings emphasize the existence of a fine and dynamic interplay between cortical GABAergic circuits, integrity of PNN CSPGs, and spontaneous plasticity of the adult-injured CST. Unlike previous observations in sensory cortical areas (Pizzorusso et al. 2002), intracortical ChABC delivery after SCI perturbs anatomical and functional CST reorganization, suggesting that spatial changes in cortical PNN integrity must be tightly controlled for meaningful reorganization of cortical motor output.

## Electronic supplementary material

Below is the link to the electronic supplementary material.

Supplementary material 1 (PDF 790 kb)
