# Supplementary material for: Integrity of cortical perineuronal nets influences corticospinal tract plasticity after spinal cord injury
Source: Brain Struct Funct. 2014 Jan 31;220(2):1077–91. doi: 10.1007/s00429-013-0701-9 (PMC4341008; doi:10.1007/s00429-013-0701-9)
Supplement: Supplementary file 1 — Supplementary material 1 (PDF 790 kb) [file 429_2013_701_MOESM1_ESM.pdf]

SUPPLEMENTARY MATERIAL

Brain Structure and Function

Integrity of Cortical Perineuronal Nets Influences Corticospinal Tract Plasticity after Spinal Cord Injury

Orlando C.[1] and Raineteau O.[1]

[1] Brain Research Institute, University of Zurich / ETH, Winterthurerstrasse 190, 8057, Zurich, Switzerland. Tel. +41 44 635 3288. Fax +41 44 635 3303. raineteau@hifo.uzh.ch

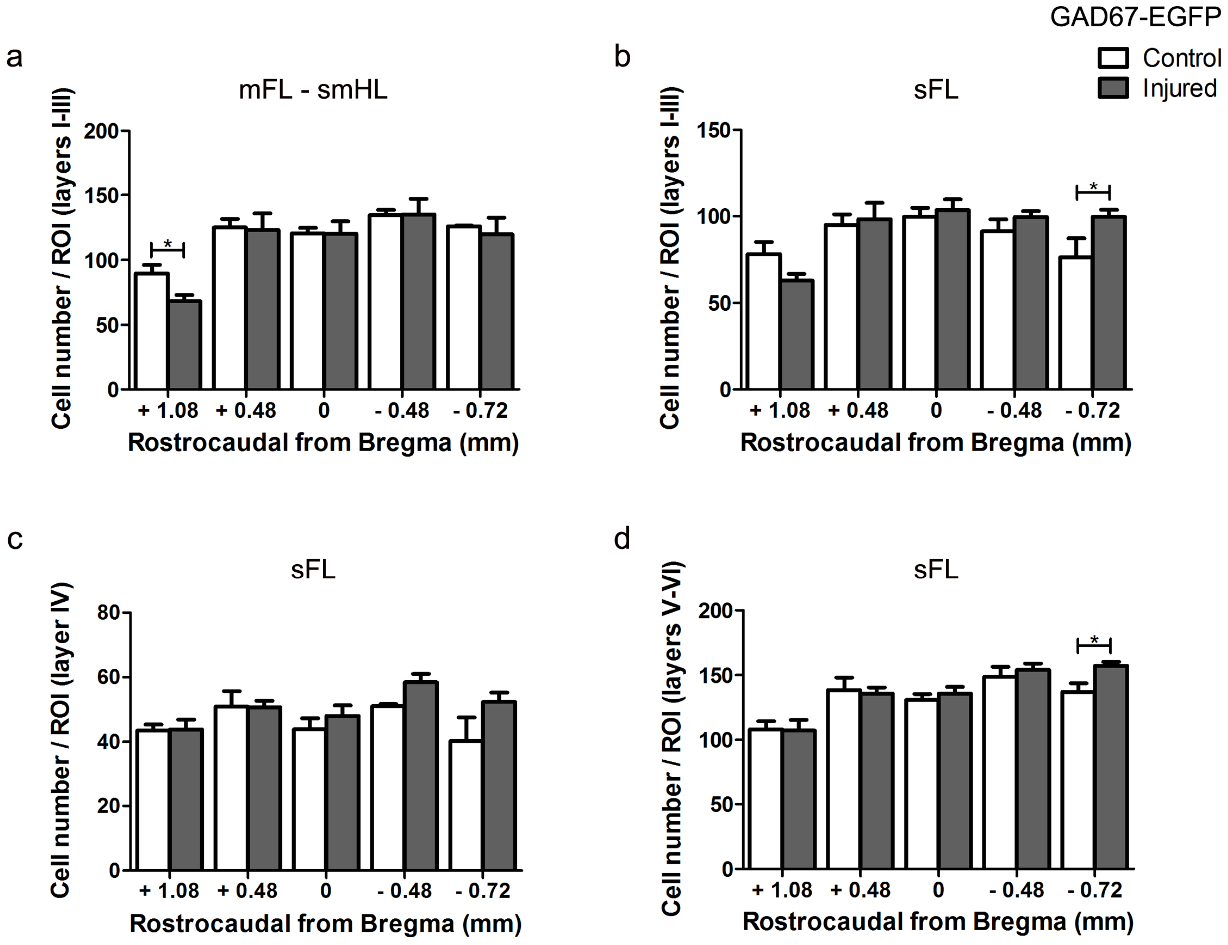

Online Resource 1. Distribution pattern of GAD67-GFP+ cells. **a**, The total number of GAD67-GFP+ cells was significantly decreased in layers I-III of the mFL (+ 1.08 rostrocaudal; 1.37 mediolateral, p = 0.027) of injured (grey bars) compared to control (white bars) mice. **b**-**d**, The total number of GAD67-GFP+ cells was significantly increased in layers I-III (- 0.72 rostrocaudal; 2.5 mediolateral; p = 0.037, **b**) and layers V-VI (p = 0.011, **d**) of the sFL of injured (grey bars) compared to control (white bars) mice. No significant changes were detected in layer IV (**c**). * $p < 0.05$, Student's t test.

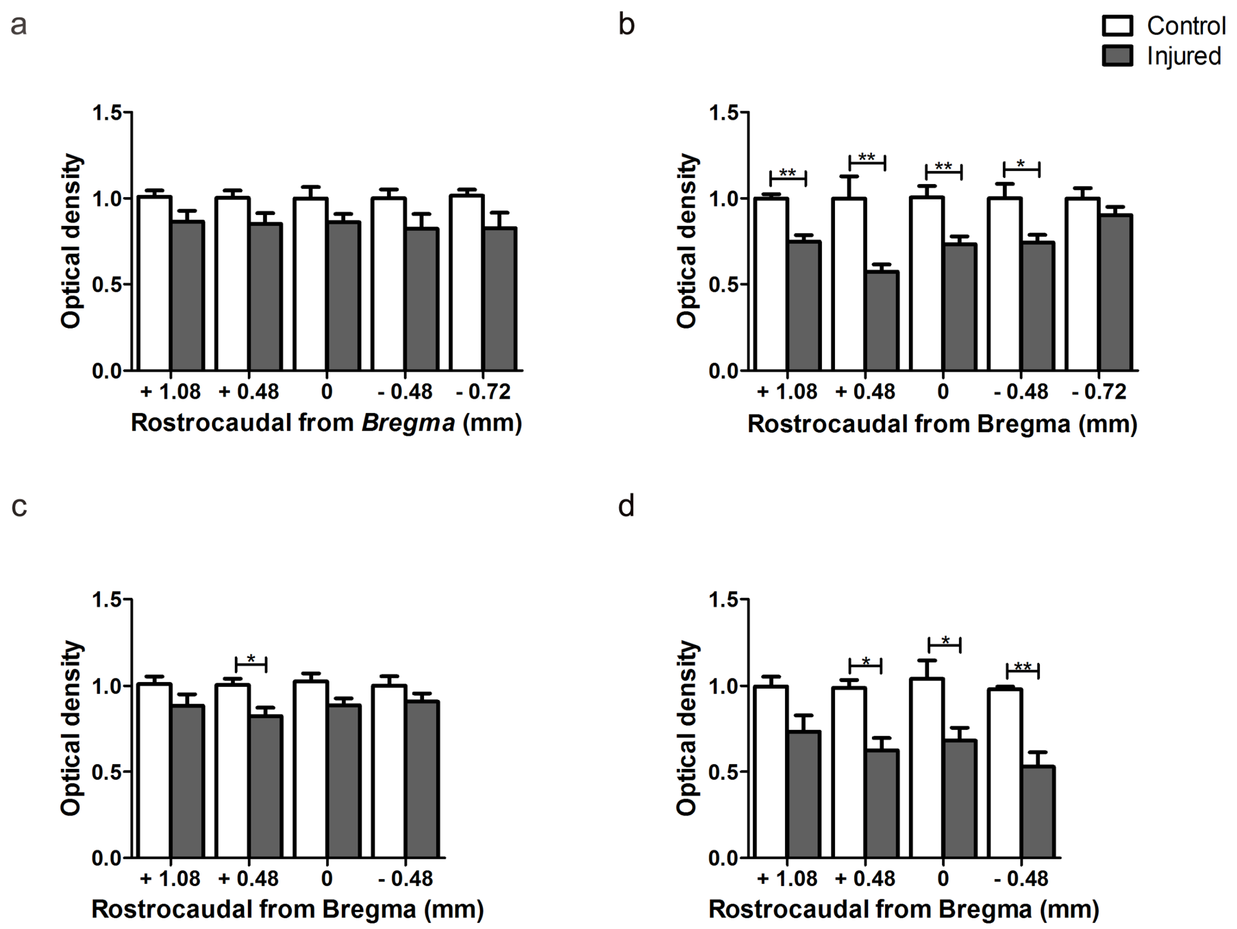

Online Resource 2. **a-b**, Single cell optical density of GAD67-GFP in the whole population of PV+ (**a**) and PV- (**b**) cells in layer V of injured (gray bars) compared to control (white bars) mice. **c-d**, Single cell optical density of GAD67-GFP in cells surrounded by WFA+ PNNs. GAD67-GFP expression was measured in PV interneurons that had maintained (WFA+/PV+, **c**) or downregulated (WFA+/PV-, **d** and Fig. 3a and b) PV in layer V of injured (gray bars) compared to control (white bars) mice. WFA+/GAD67-GFP-/PV- cells were excluded from the analysis. * $p < 0.05$, ** $p < 0.01$, Student's t test.

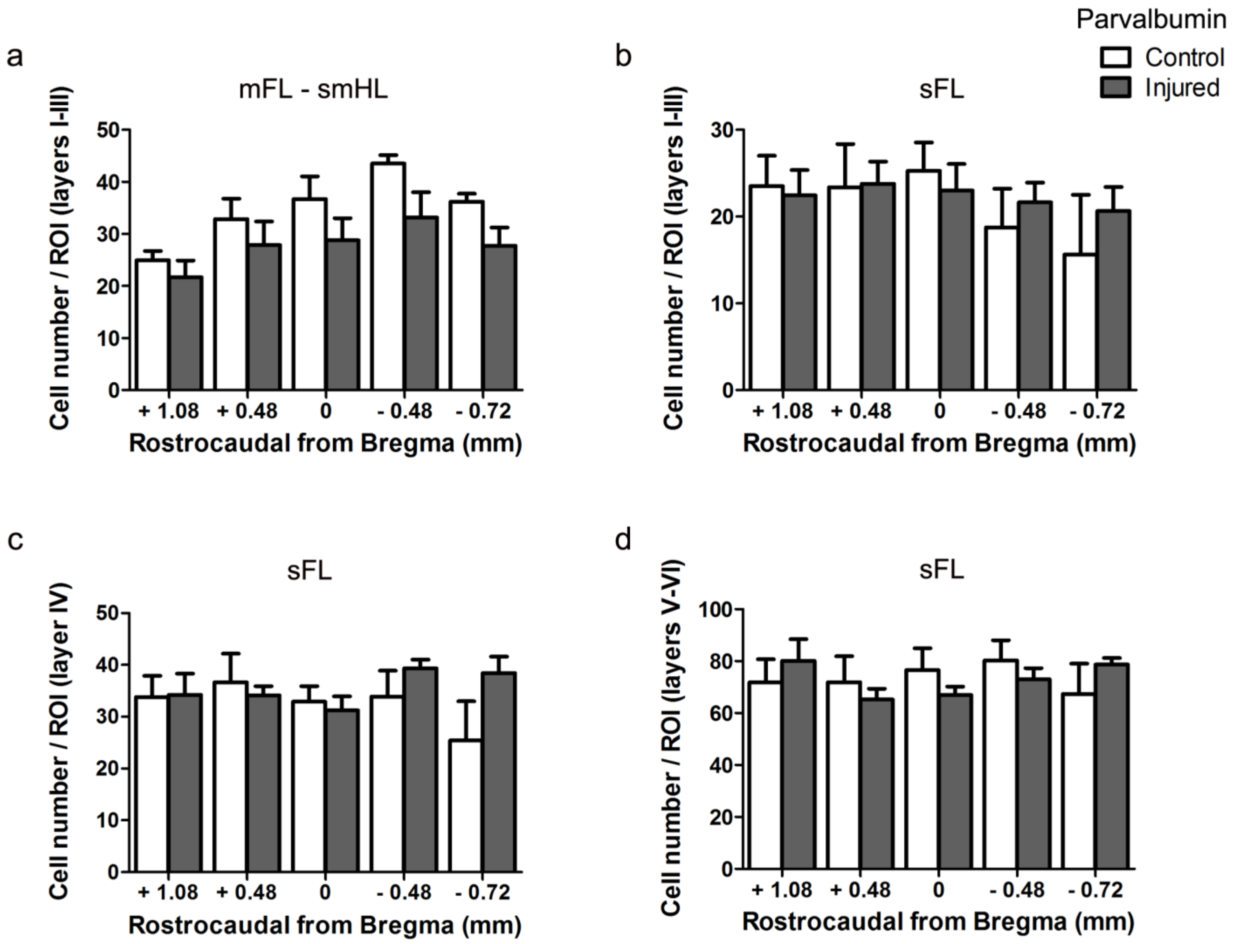

Online Resource 3. Distribution pattern of PV+ cells. **a-d**, The total number of PV+ cells was not significantly changed either in the upper layers (I-III) of the mFL and smHL or in all the layers of the sFL cortex. Student’s t test.

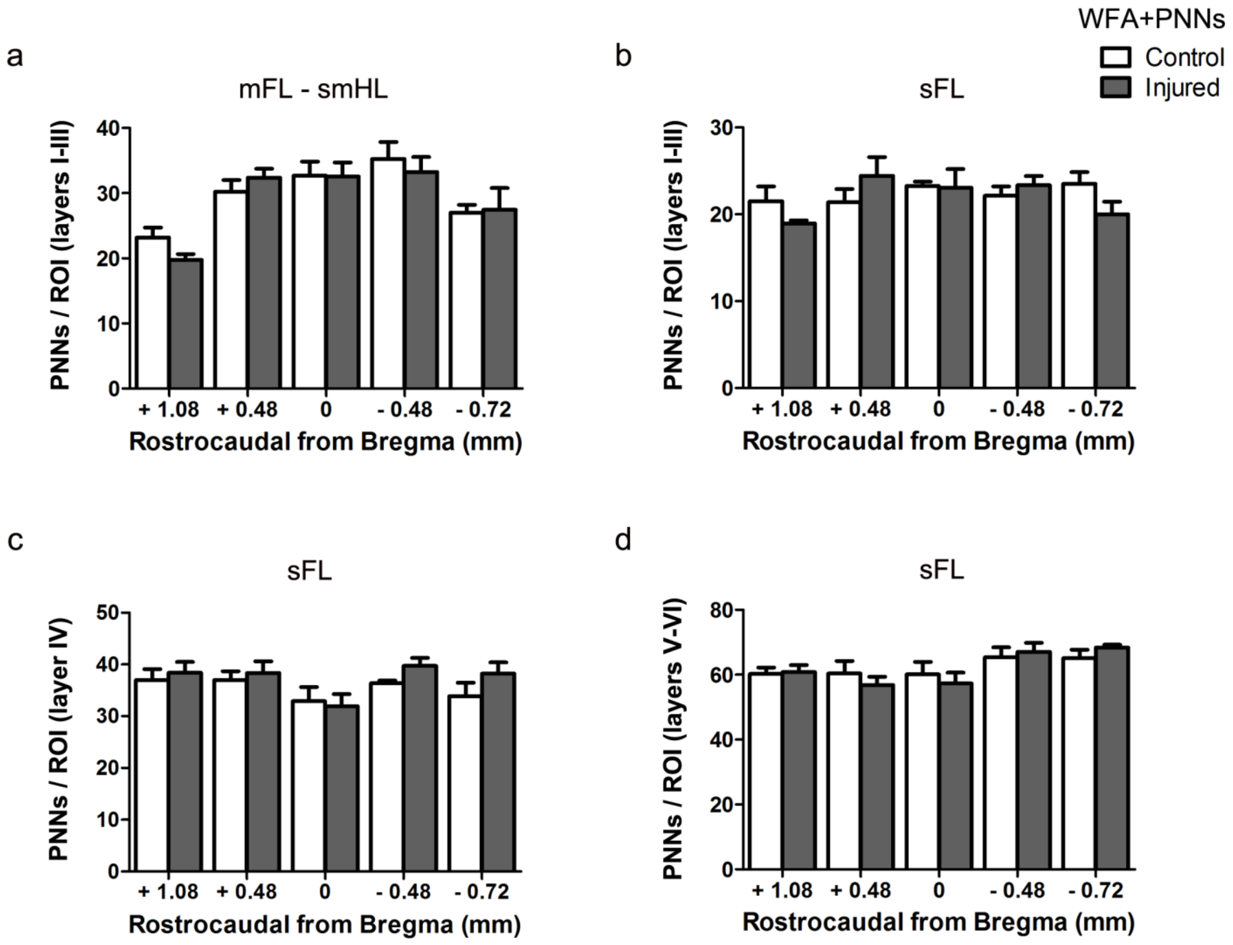

Online Resource 4. Distribution pattern of WFA+ PNNs. **a**-**d**, The total number of WFA+ PNNs was not significantly changed either in the upper layers (I-III) of the mFL and smHL or in all the layers of the sFL cortex. Student’s t test.

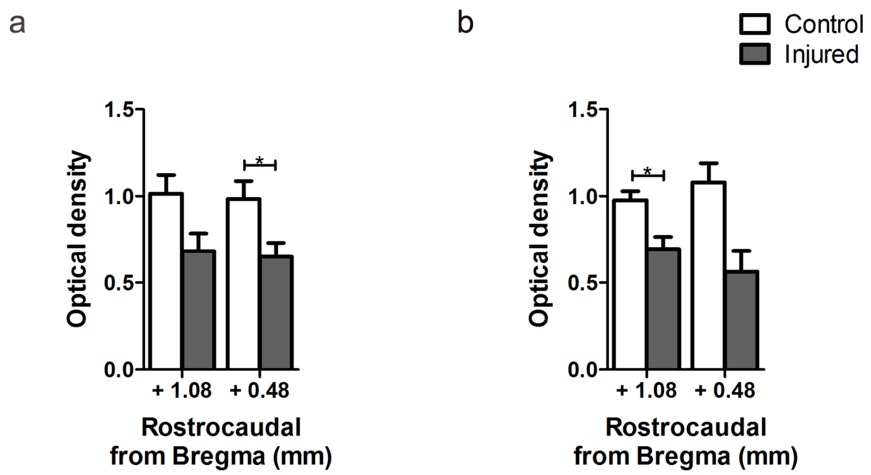

Online Resource 5. Optical density measurements of WFA+ PNNs at the transition region between the mFL and smHL cortex. Single PNNs were measured around GAD67-GFP+/PV+ (**a**) and GAD67-GFP+/PV- (**b**) cells in layer V of injured (gray bars) compared to control (white bars) mice. * $p < 0.05$, Student's t test.
